# How to diagnose plantaris tendon involvement in midportion Achilles tendinopathy - clinical and imaging findings

**DOI:** 10.1186/s12891-016-0955-5

**Published:** 2016-02-24

**Authors:** Lorenzo Masci, Christoph Spang, Hans T. M. van Schie, Håkan Alfredson

**Affiliations:** Pure Sports Medicine Clinic, London, UK; Department of Integrative Medical Biology (IMB), Anatomy Section, Umea University, 90187 Umea, Sweden; Department of Scientific Research, UTC Imaging, Stein, The Netherlands; Department of Surgical and Perioperative Sciences, Sports Medicine Unit, Umea University, Umea, Sweden; ISEH, University College London Hospitals, London, UK

**Keywords:** Achilles tendinopathy, Plantaris tendon, Ultrasound Tissue Characterisation, Ultrasound, Medial pain

## Abstract

**Background:**

The purpose of this investigation was to evaluate if clinical assessment, Ultrasound + Colour Doppler (US + CD) and Ultrasound Tissue Characterisation (UTC) can be useful in detecting plantaris tendon involvement in patients with midportion Achilles tendinopathy.

**Methods:**

Twenty-three tendons in 18 patients (14 men, mean age: 37 years and 4 women: 44 years) (5 patients with bilateral tendons) with midportion Achilles tendinopathy were surgically treated with a scraping procedure and plantaris tendon removal. For all tendons, clinical assessment, Ultrasound + Colour Doppler (US + CD) examination and Ultrasound Tissue Characterisation (UTC) were performed.

**Results:**

At surgery, all 23 cases had a plantaris tendon located close to the medial side of the Achilles tendon. There was vascularised fat tissue in the interface between the Achilles and plantaris tendons. Clinical assessment revealed localised medial activity-related pain in 20/23 tendons and focal medial tendon tenderness in 20/23 tendons. For US + CD, 20/23 tendons had a tendon-like structure interpreted to be the plantaris tendon and localised high blood flow in close relation to the medial side of the Achilles. For UTC, 19/23 tendons had disorganised (type 3 and 4) echopixels located only in the medial part of the Achilles tendon indicating possible plantaris tendon involvement.

**Conclusions:**

US + CD directly, and clinical assessment indirectly, can detect a close by located plantaris tendon in a high proportion of patients with midportion Achilles tendinopathy. UTC could complement US + CD and clinical assessment by demonstrating disorganised focal medial Achilles tendon structure indicative of possible plantaris involvement.

## Background

Chronic pain symptoms from the Achilles midportion, referred to as tendinopathy, is a disabling condition in both active and non-active individuals [1]. Despite recent research advances, the pathogenesis remains elusive [2, 3] and includes overuse and compressive factors.

Novel surgical treatments targeting the nerve and blood vessel-rich peritendinous tissue on the ventral side of the Achilles tendon (e.g. the minimally-invasive scraping procedure) [4] have shown good results [4, 5]. Post-surgical outcomes revealed a sub-group of patients who were non-responsive to scraping. These patients often had medially- located tendon pain and a thickened plantaris located close to the medial Achilles tendon. Subsequent removal of this thickened plantaris has shown to improve outcomes [5–8]. Excised plantaris tendons were macroscopically found to be thicker than normal plantaris tendons [6], and histological analyses showed tendinosis-like changes with a similar matrix degradation as described for midportion Achilles tendinopathy [9, 10]. Despite these recent observations of plantaris tendon involvement in a subset of midportion Achilles tendinopathy, there is a paucity of information regarding diagnosis of plantaris involvement using clinical and imaging findings.

Ultrasound + Colour Doppler (US + CD) is commonly used for diagnosing tendinosis-like changes in tendons [11, 12]. Although there is evidence that the plantaris tendon can be detected by ultrasound [13], there are, to the best of our knowledge, no studies on humans comparing macroscopic and ultrasound findings in patients with midportion Achilles tendinopathy and potential plantaris tendon involvements. A novel imaging modality called Ultrasound Tissue Characterisation (UTC) has recently been introduced to visualise Achilles tendon structure and to quantify tendon matrix integrity [14, 15]. Via contiguous transverse ultrasound images that are collated at even distances, a 3D volume block of ultrasound data is created. UTC algorithms objectively quantify grey-scale changes into 4 different echo-types related to matrix integrity (intact to completely disorganised fibrillar matrix). Currently, there are no studies that have investigated the use of UTC in diagnosing plantaris tendon involvement in midportion Achilles tendinopathy.

With the aforementioned knowledge about plantaris tendon involvement in a subset of midportion Achilles tendinopathy, it is of interest to evaluate methods for diagnosis. The primary aim of this study was to evaluate if clinical assessment, US + CD and UTC could predict the presence of a plantaris tendon located close to the medial side of the Achilles tendon in patients with chronic painful midportion Achilles tendinopathy. Surgical exploration was used to verify the location/position of the plantaris tendon in relation to the Achilles tendon.

## Methods

### Patients

Twenty-three tendons from 18 consecutive patients (14 men, 4 women), mean age of 40 years (range 25–60 years) with painful midportion Achilles tendinopathy referred for surgical opinion were included. No cases had had pervious surgical treatment or inflammatory diseases. Duration of symptoms ranged from 4 to 120 months.

Sixteen patients had failed a tendon loading program. Nine patients had been treated with injection therapy (high volume saline alone, 3; high volume saline + marcaine + cortisone, 4; cortisone alone, 2). Seven patients were elite athletes (track and field, 3; football, 1; rugby, 1; acrobatics, 1; boxing 1) whereas eleven patients were non-elite (running, 10; football, 1).

### Pre-surgical examination

#### Clinical assessment

Patients were asked specifically about location of tendon pain. Clinical examination was performed in a prone position with the feet hanging freely over the edge of the examination bed and evaluated by the same examiner (HA). Location of tenderness on tendon palpation was noted.

### US + CD examination

Ultrasound examinations were performed in a prone position with the feet hanging freely over the edge of the examination bed. A high-resolution greyscale Ultrasound (US) and Colour + Doppler (CD) (Antares-Siemens, Germany) with a linear multifrequency (8–13 MHz) probe was used. Colour Doppler settings were standardised with a gain of 68 dB, sensitivity of 8 cm/s and a pulse repetition frequency of 1250Hz. Achilles tendon thickness, structure and blood flow was assessed by the same examiner (HA) with over 15 years experience in diagnostic musculoskeletal ultrasound. Tendon structure was defined as being normal or irregular and hypoechoic. Furthermore, the location of the hypoechogenicity (medial, ventral, lateral) was noted. The examined Achilles tendons were assessed as having normal or high blood flow. Tendons with “normal blood flow” had no detectable blood flow in the sagittal or axial plane, whereas “high blood flow” tendons exhibited at least one region with localised high blood flow in the sagittal and axial plane. Careful examination of the medial side of the Achilles tendon, starting proximally in the mid calf and moving distally, was performed to look for the plantaris tendon.

### Ultrasound Tissue Characterisation (UTC)

When taking UTC scans, patients were positioned prone on the examination table with the affected ankle caudal to the edge of the examination bed. The examiner’s knee was then pushed against the forefoot of the patients in order to achieve maximal passive dorsiflexion. Scans were collected in a distal to proximal direction, and were performed by the same experienced examiner (LM). More practical details are described in [7].

UTC algorithms were applied to quantify the stability of grey scale levels of corresponding pixels in contiguous images over 25 images. Previous studies using histopathological tissue specimen as reference have shown that the stability of grey scale levels strongly correlates with the architecture and integrity of the tendon matrix [16–19]. Furthermore, UTC has demonstrated high intra- and inter-rater reliability [14, 15].

Validated UTC algorithms can discriminate 4 different echo-types, related to matrix integrity, and is visualised as four different colours. Type I pixels (green) represent intact, continuous and aligned collagen bundles (fibres and fasciculi); type II (blue) indicates less continuous and/or more swollen and/or more wavy collagen bundles (fibres and fasciculi); type III (red) is generated by disintegration with tendon tissue replaced by mainly disorganised, fibrillar matrix; type IV (black) is generated by complete disintegration with tendon tissue replaced by an amorphous matrix and fluid.

Regions with disintegration (echopixels type III and IV) within the midportion of the Achilles tendon were identified and recorded according to site within the midportion of the tendon (superficial, ventral, medial or lateral).

### Surgical treatment

Surgery was undertaken 24 h after clinical assessment, US + CD and UTC examinations. Under local anesthesia [6], the medial aspect of the Achilles tendon was visualised via a minor longitudinal incision on the medial side of the Achilles tendon midportion. For determination of the presence and exact location of a plantaris tendon, the medial side of the Achilles tendon was carefully inspected. When a plantaris tendon was detected, the location in relation to the medial aspect of the Achilles tendon was documented with photographs. The surgical treatment consisted of release of the plantaris tendon followed by excision distally from the calcaneal insertion and proximally at a level slightly above the distal medial soleus musculotendinous junction. In addition, a ventral tendon scraping procedure was performed in the regions corresponding Ultrasound + Doppler-verified high blood flow on the ventral side of the Achilles tendon [4]. The length of the longitudinal incision was 1–2 cm. All operations were performed by the same surgeon (HA).

### Ethics

The study was approved by University of Queen Mary ethics committee as part of a prospective study on all surgically-treated Achilles tendons at Pure Sports Medicine Clinic in London, UK. All participants provided informed consent prior to participation.

## Results

### Macroscopical (in-wound) surgical findings

**Fig. 1:**
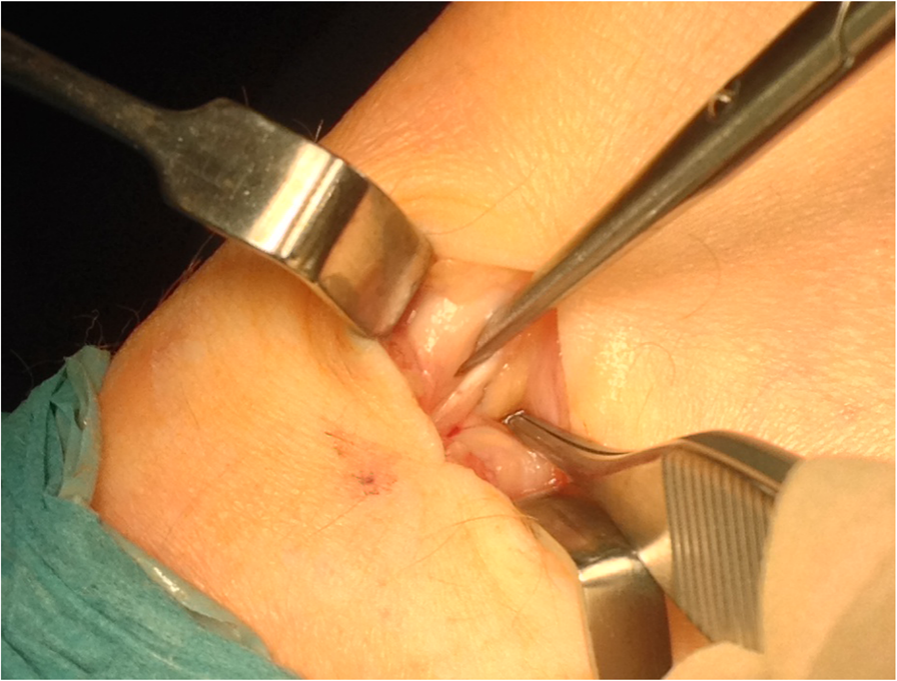
Thickened plantaris tendon in close relationship to the medial Achilles tendon found during surgical exploration

In 23/23 surgical explorations, the plantaris tendon was identified. For twenty-one Achilles tendons, a plantaris tendon was found in close relation to the medial side of the Achilles midportion (Fig. 1). There was richly vascularised fat tissue between the Achilles and plantaris tendons. For two Achilles tendons, a plantaris tendon was invaginated into the medial Achilles tendon. There was no significant correlation between duration of symptoms and severity of macroscopic pathological changes or imaging findings.

### Clinical assessment

There was medial activity-related tendon pain in 20/23 tendons. Three patients had medial and lateral activity-related tendon pain. There was tenderness on the medial side of the tendon in 20/23 tendons. In 3/20 tendons, there was medial and lateral tendon tenderness.

### US + CD

**Fig. 2:**
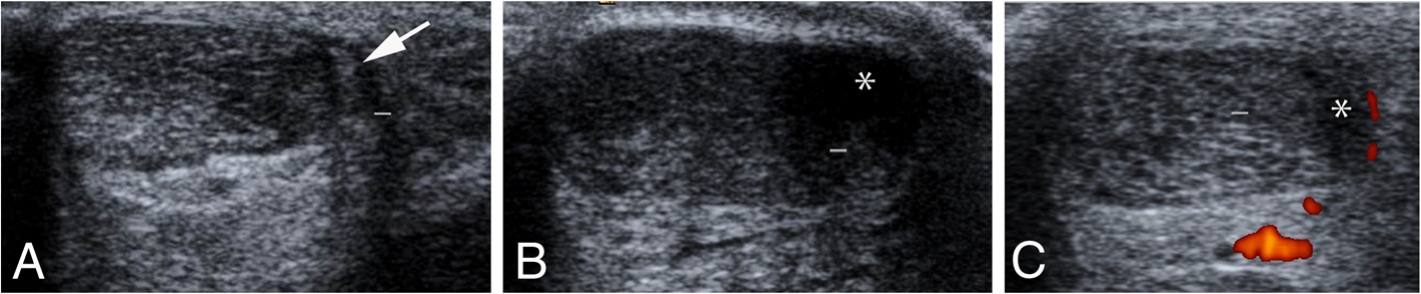
US+CD results: Occurrence of a plantaris tendon-like structure (arrow) in close relation to the medial side of the Achilles tendon (**a**). Localised medial hypoechoic changes (**b**, **c**; asterisk) and high blood flow close to the medial side of the Achilles and deeper to the ventral side (**c**)

In 20/23 tendons, a plantaris tendon-like structure was visible in close relation to the medial side of the Achilles tendon (Fig. 2a). In three ultrasound cases, a plantaris tendon could not be detected. Localised medial hypoechoic changes (Fig. 2b, c) and localised high blood flow were detected in 20/23 Achilles tendons (Fig. 2c). Medial and lateral hypoechoic changes and high blood flow were detected in 3/23 tendons. No tendons demonstrated isolated localised lateral hypoechoic changes and high blood flow.

### UTC

**Fig. 3:**
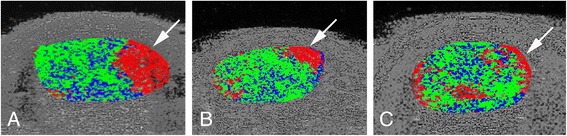
UTC results: Frequent occurrence of focal disorganised type 3 (red) and some type 4 (black)) echopixels in the medial part (arrows) of the midportion of the Achilles tendon (**a**, **b**). Less frequent observation of general disorganised (type 3 and 4) echopixels in both the medial and lateral parts of the midportion of the Achilles tendon (**c**)

In 19/23 tendons, there were focal disorganised (type 3 and 4) echopixels in the medial part of the Achilles midportion (Fig. 3a, b). In 4/23 tendons, there were disorganised (type 3 and 4) echopixels in both medial and lateral parts of the midportion of the Achilles tendon (Fig. 3c).

## Discussion

In this study on surgically treated patients with midportion Achilles tendinopathy, surgical exploration of all cases demonstrated a plantaris tendon located close to, and in 2 cases invaginated into, the medial side of the Achilles midportion. Clinical assessment reported medial tendon pain and tenderness in all 23 tendons, and US + CD suspected plantaris tendon involvement in 20/23 tendons via a direct visualisation of a thickened plantaris tendon medial to the midportion of the Achilles tendon. Using UTC, all cases demonstrated disorganised changes in the medial Achilles mid-portion. These changes may represent an indirect sign of plantaris tendon involvement via compression of the plantaris on the medial aspect of the Achilles tendon.

The plantaris tendon is increasingly recognised as an important factor in midportion Achilles tendinopathy. Recent cadaveric [20], histological [9, 10], and clinical/surgical [5–8] studies lend support to the theory that the plantaris tendon may be involved in the pathogenesis and/or the aetiology of midportion Achilles tendinopathy. It would be reasonable to imply that there is a sub-set of patients with plantaris-associated midportion Achilles tendinopathy.

We found that in all 23 tendons, there was medial tenderness during clinical examination. In addition, 15/18 patients (20/23 tendons) reported focal activity-related pain on the medial side of the Achilles tendon. Tendon tenderness and pain are considered relatively non-specific findings; however, the localisation of pain and tenderness at the medial Achilles tendon could be an important clinical marker of plantaris tendon involvement in midportion Achilles tendinopathy and may warrant further investigation.

For US + CD, a majority of tendons revealed an obvious tendon-like structure that made the diagnosis of a plantaris tendon involvement relatively straightforward. For some tendons, there was a less distinct tendon-like structure. The variability of the appearance of a plantaris tendon is not surprising as a recent study on 107 cadavers found a significant variability in the appearance, size and positions of the plantaris tendon [20]. In addition to the visualisation of the plantaris tendon, US + CD revealed hypoechoic regions localised on the medial Achilles tendon midportion in all tendons. As the plantaris was located close to the medial side of the Achilles tendon, these hypoechoic medial Achilles changes suggest a possible direct association between the location of the plantaris tendon and localised tendon changes. This may theoretically be due to compressive forces by the plantaris tendon onto the medial Achilles tendon. It has been hypothesised that compression might be the trigger in the interaction of plantaris-induced Achilles tendinopathy [6, 21]. There are a few observations that may support this hypothesis. First, plantaris tendons are known to be stronger and stiffer than the Achilles tendon [22] and may thus apply compressive and/or shearing forces onto the Achilles tendon in cases with close positioning between the tendons. Second, recent findings demonstrating gradual improvement of Achilles tendon structure after surgical removal of the plantaris tendon further support the compression theory [7]. More studies are, however, needed to fully explain the mechanisms between the two tendons in this condition.

Ultrasound tissue characterisation (UTC) objectively quantifies grey-scale tendon matrix changes and has been shown to be reliable and valid [14–19]. In this study, the UTC examination showed that in 19/23 tendons, there were marked focal changes in the medial aspect of the Achilles tendon alone; and in another 4/23 tendons, there were focal changes in both the medial and lateral side of the Achilles tendon midportion. In this study, it was not possible to accurately visualise the plantaris tendon separately from the medial Achilles. There could be many reasons as to why the plantaris is difficult to visualise with UTC. One reason may be related to the presence of co-existing tendinopathy within the plantaris tendon. Recent studies revealed tendinosis-related changes in plantaris tendons associated with midportion Achilles tendinopathy [9, 10]. As the plantaris tendon was positioned very close to the medial Achilles tendon, the presence of tendinosis-changes in both structures may make the distinction between Achilles and plantaris structures challenging on UTC. Consequently in this study, UTC was valuable at indirectly suspecting plantaris involvement by demonstrating disorganised changes in the medial aspect of the Achilles tendon. These changes may be related to compression or mechanical interference of the plantaris on the medial Achilles mid-portion. Therefore, although UTC may not directly visualise a plantaris tendon, it may complement clinical presentation and direct visualisation of the plantaris tendon on US + CD and assist in diagnosing a subset of mid-portion Achilles tendinopathy with plantaris tendon involvement.

One limitation with this study was the relatively small sample size. Studies on a larger sample size are in progress. In addition, this study failed to examine the role of MRI in visualising a plantaris tendon and possible involvement in mid-portion Achilles tendinopathy. However, it is the authors’ clinical experience that MRI is less sensitive at detecting a plantaris tendon compared to US + CD. As such, MRI scan is not routinely performed by the authors in the assessment of Achilles tendinopathy. Studies comparing MRI with US + CD and UTC would complement this study.

## Conclusion

In conclusion, in patients with midportion Achilles tendinopathy, medial tenderness and activity related medial pain might indicate plantaris tendon involvement. US + CD can directly visualise the plantaris tendon in a high proportion of the patients, and UTC seems to have potential to indirectly detect a plantaris tendon located close to the medial Achilles in a majority of the patients. While not essential in diagnosing plantaris involvement, UTC may complement clinical assessment and US + CD in confirming plantaris-involvement, which may significantly change clinical management in patients with mid-portion Achilles tendinopathy.

## Abbreviations

UTC: Ultrasound Tissue Characterisation
US: Ultrasound
CD: Colour Doppler
LM: Lorenzo Masci
CS: Christoph Spang
HS: Hans van Schie
HA: Håkan Alfredson
